# Supplementary material for: Surface protein profiling of prostate-derived extracellular vesicles by mass spectrometry and proximity assays
Source: Commun Biol. 2022 Dec 22;5:1402. doi: 10.1038/s42003-022-04349-x (PMC9780212; doi:10.1038/s42003-022-04349-x)
Supplement: Supplementary file 2 — Supplementary Information [file 42003_2022_4349_MOESM2_ESM.pdf]

Surface protein profiling of prostate-derived extracellular vesicles by mass spectrometry and proximity assays

Ehsan Manouchehri Doulabi[1¥], Claudia Fredolini[1¥], Radiosa Gallini[1], Liza Löf[1], Qiujin Shen[1], Ryoyo Ikebuchi[1,2], Louise Dubois[3], Alireza Azimi[1], Olivier Loudig[4], Susanne Gabrielsson[5], Ulf Landegren[1], Anders Larsson[3], Jonas Bergquist[6] and Masood Kamali-Moghaddam[1]*

[1]Department of Immunology, Genetics & Pathology, Science for Life Laboratory, Uppsala University, Uppsala, Sweden.

[2]JSPS Overseas Research Fellow, Japan Society for the Promotion of Science, Japan.

[3]Department of Medical Sciences, Clinical Chemistry, Uppsala University, Uppsala, Sweden.

[4]Center for Discovery and Innovation, Hackensack Meridian Health, New Jersey, USA.

[5]Division of Immunology and Allergy, Department of Medicine, Karolinska Institutet, Solna, Sweden.

[6]Department of Chemistry-BMC, Analytical Chemistry, Uppsala University, Uppsala, Sweden.

[¥] These authors contributed equally to this work

*To whom correspondence should be addressed. Email: masood.kamali@igp.uu.se

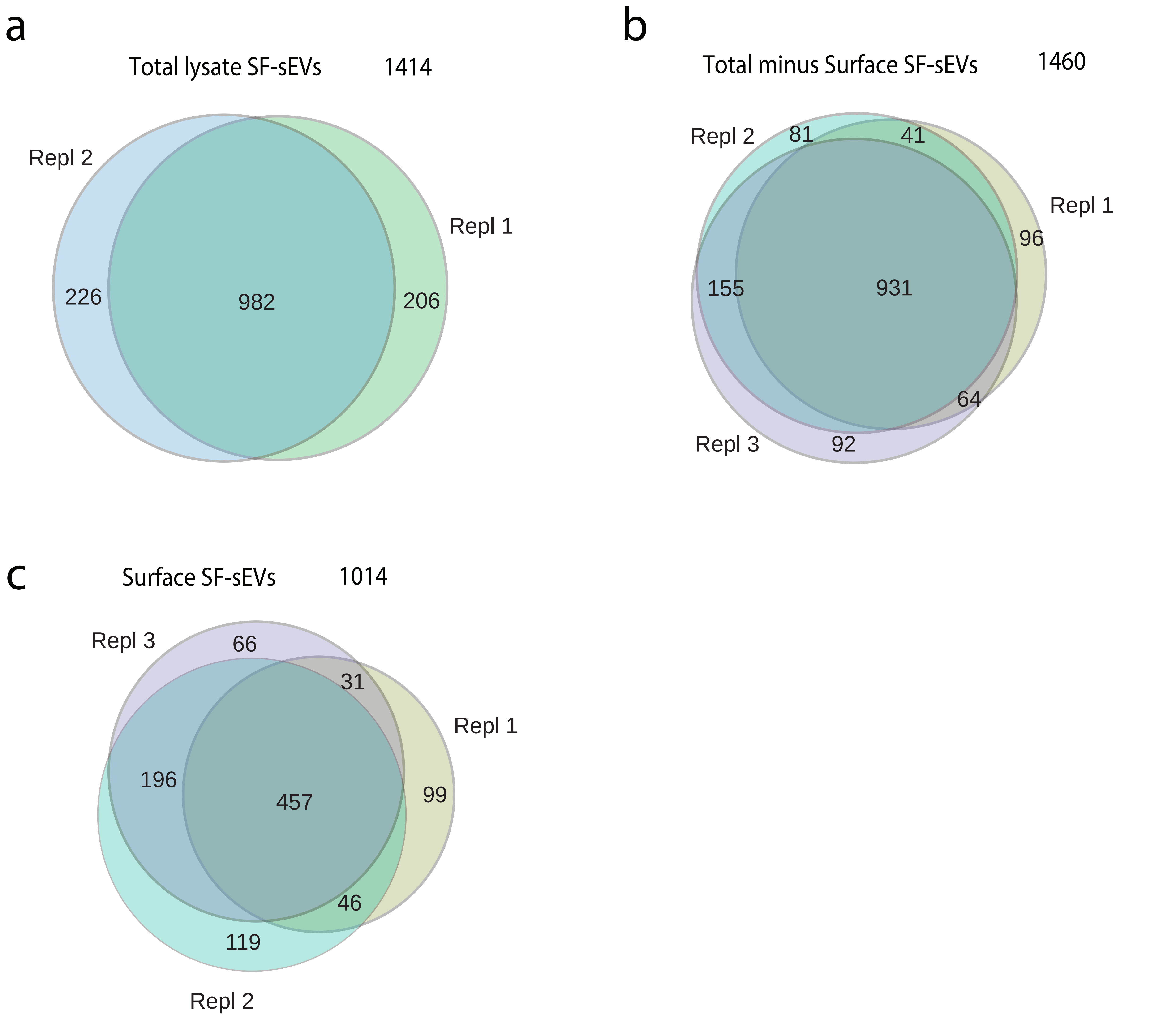
a
Total lysate SF-sEVs
1414
Repl 2
Repl 1
226
982
206
b
Total minus Surface SF-sEVs
1460
Repl 2
81
41
Repl 1
96
155
931
64
Repl 3
92
c
Surface SF-sEVs
1014
Repl 3
66
31
Repl 1
196
457
99
46
119
Repl 2

Supplementary Figure 1. Reproducibility of identified proteins.

Proportional area Venn diagrams of identified SF-sEVs proteins in (a) two replicates samples of Total lysate and three replicates samples of (b)Total-Surface and (c) Surface.

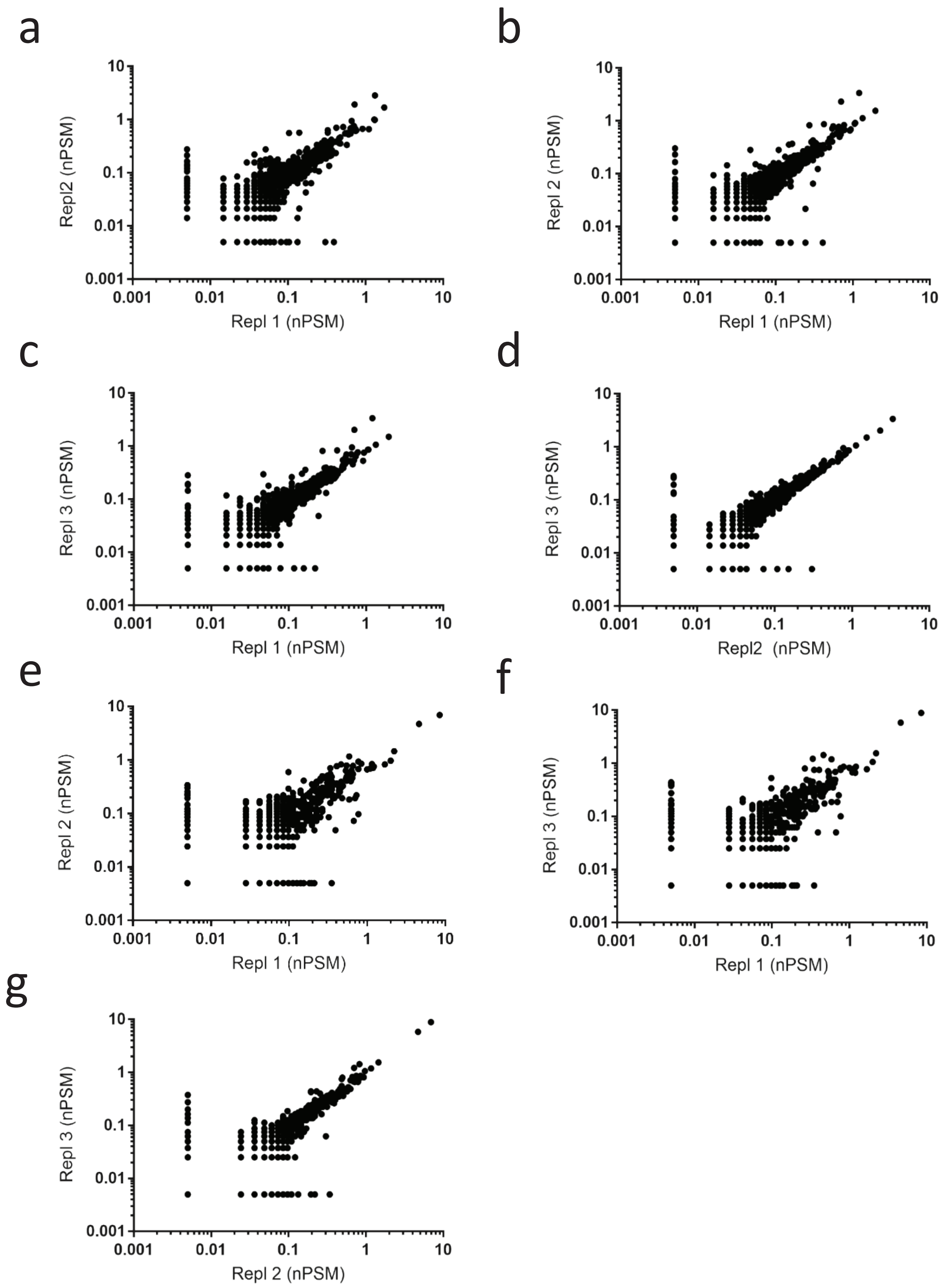
a
Repl2 (nPSM)
Repl 1 (nPSM)
b
Repl 2 (nPSM)
Repl 1 (nPSM)
c
Repl 3 (nPSM)
Repl 1 (nPSM)
d
Repl 3 (nPSM)
Repl2 (nPSM)
e
Repl 2 (nPSM)
Repl 1 (nPSM)
f
Repl 3 (nPSM)
Repl 1 (nPSM)
g
Repl 3 (nPSM)
Repl 2 (nPSM)
10
1
0.1
0.01
0.001

Supplementary Figure 2. Reproducibility of numbers of protein identifications.

Correlation between relative protein abundance (nPSMs) in replicates SF-sEVs samples of “Total lysate” (a), “Total minus Surface” (b, c, d) and “Surface” (e, f, g). Pearson’s r were respectively: (a) 0.872; (b) 0.836 (c) 0.846; (d) 0.985; (e) 0.953; (f) 0.948; (g) 0.986.

a

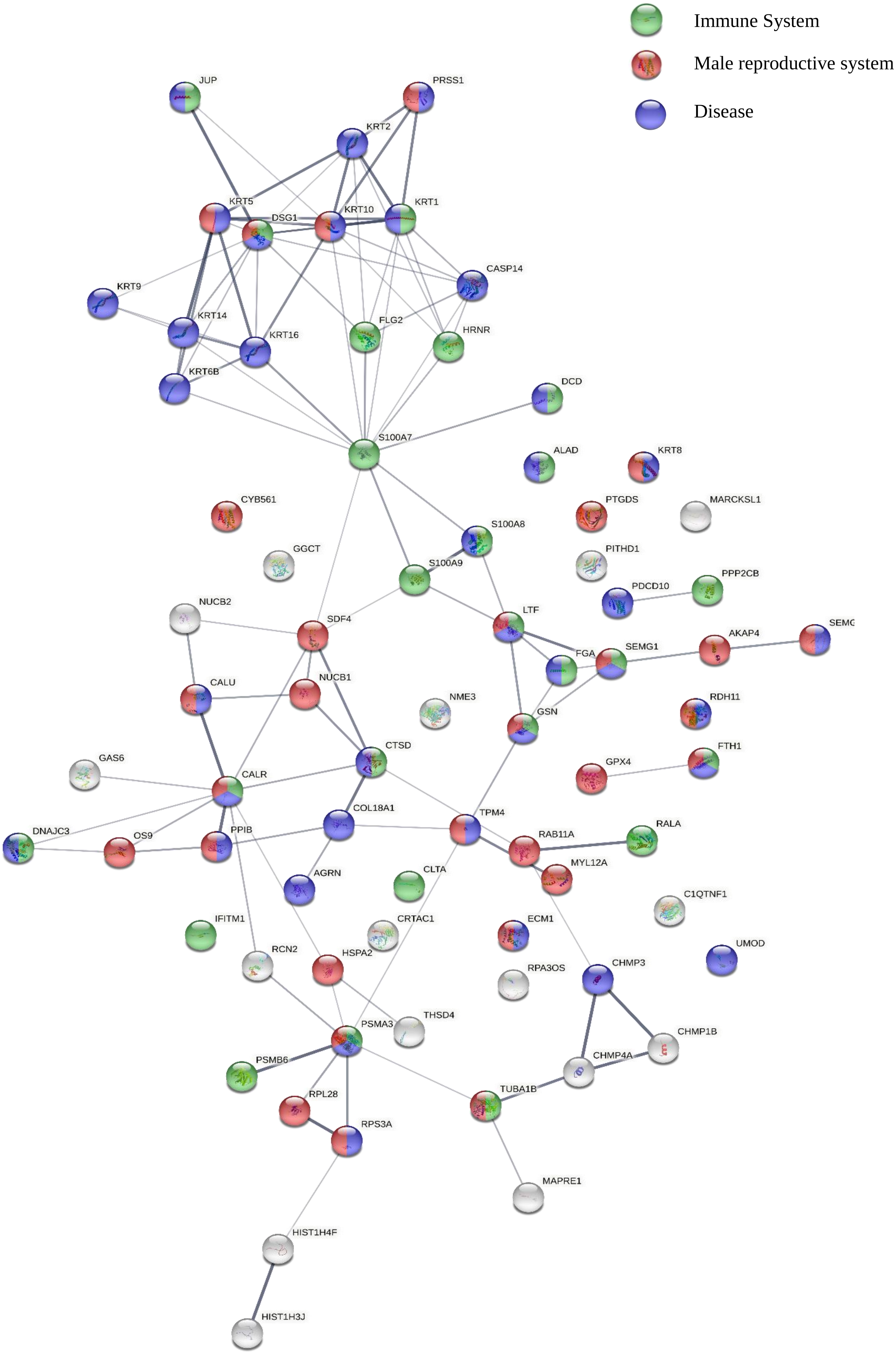

Immune System
Male reproductive system
Disease
JUP
PRSS1
KRT2
KRT5
DSG1
KRT10
KRT1
CASP14
KRT9
KRT14
KRT16
FLG2
HRNR
KRT6B
DCD
S100A7
ALAD
KRT8
CYB561
PTGDS
MARCKSL1
S100A8
GGCT
PITHD1
S100A9
PDCD10
PPP2CB
NUCB2
SDF4
LTF
SEMG
AKAP4
FGA
SEMG1
NUCB1
CALU
NME3
RDH11
GSN
CTSD
FTH1
GAS6
GPX4
CALR
TPM4
COL18A1
RALA
RAB11A
DNAJC3
OS9
PPIB
MYL12A
AGRN
CLTA
C1QTNF1
IFITM1
CRTAC1
ECM1
UMOD
RCN2
HSPA2
RPA3OS
CHMP3
THSD4
PSMA3
CHMP1B
PSMB6
CHMP4A
TUBA1B
RPL28
RPS3A
MAPRE1
HIST1H4F
HIST1H3J

b

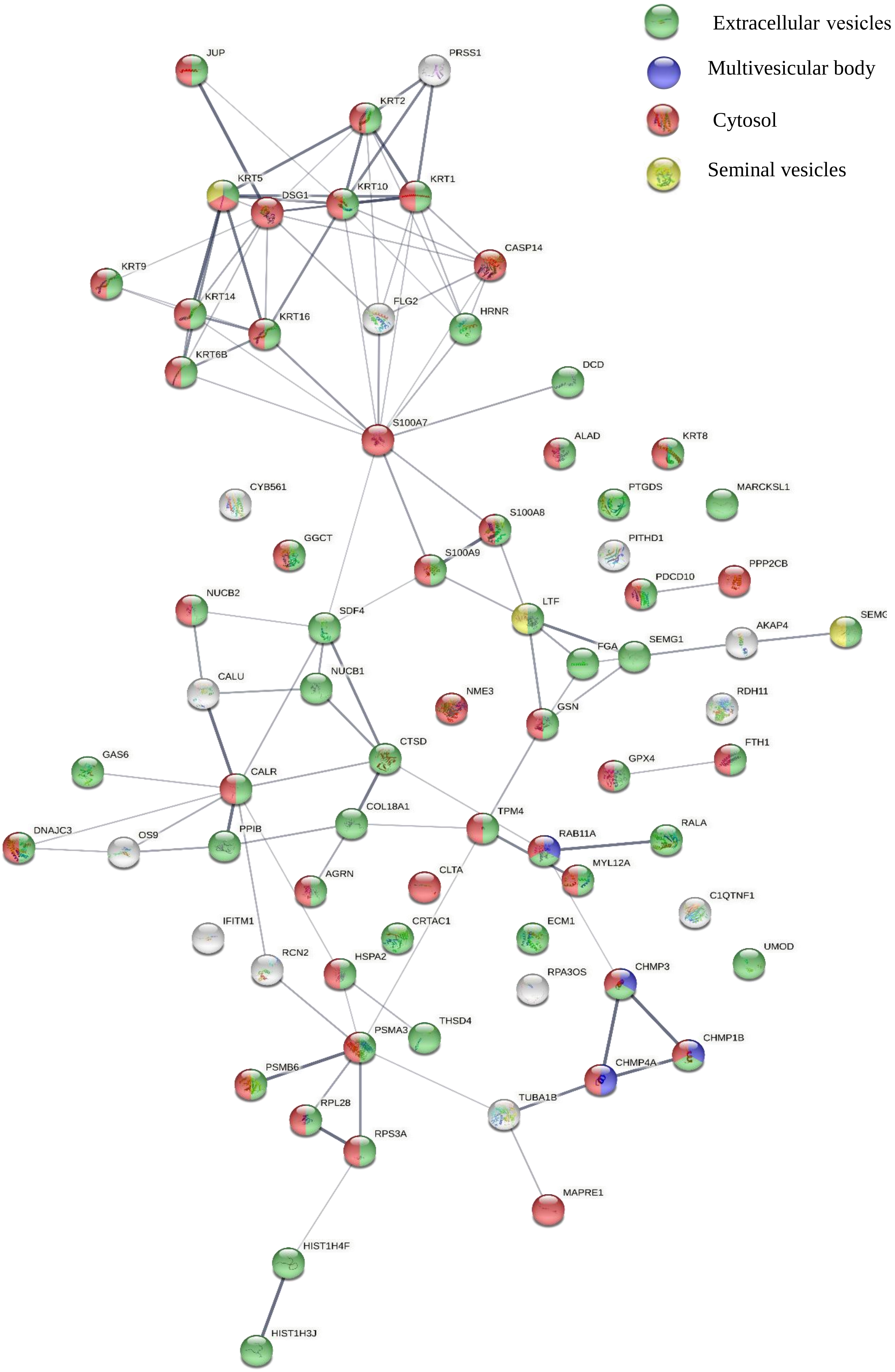

Extracellular vesicles
Multivesicular body
Cytosol
Seminal vesicles
JUP
PRSS1
KRT2
KRT5
DSG1
KRT10
KRT1
CASP14
KRT9
KRT14
FLG2
HRNR
KRT16
KRT6B
DCD
S100A7
ALAD
KRT8
CYB561
PTGDS
MARCKSL1
S100A8
GGCT
PITHD1
S100A9
PDCD10
PPP2CB
NUCB2
LTF
SDF4
FGA
SEMG1
AKAP4
NUCB1
CALU
NME3
GSN
RDH11
CTSD
GAS6
CALR
FTH1
GPX4
COL18A1
TPM4
DNAJC3
PPIB
OS9
RAB11A
RALA
MYL12A
AGRN
CLTA
C1QTNF1
IFITM1
CRTAC1
ECM1
UMOD
RCN2
HSPA2
RPA3OS
CHMP3
THSD4
PSMA3
CHMP1B
PSMB6
CHMP4A
TUBA1B
RPL28
RPS3A
MAPRE1
HIST1H4F
HIST1H3J

Supplementary Figure 3. String Graph.

The location and functional roles of the 74 Surface enriched proteins were analyzed using the STRING database. The analysis was based on seven different criteria; a) 1) play roles in immune system, 2) any putative function in male reproductive system, and 3) putative involvement in emerging of diseases. b) presence in 4) extracellular vesicles, 5) multivesicular bodies, 6) cytosol and 7) seminal vesicles. White colored dot indicates that a protein is not existed in any of selected options.

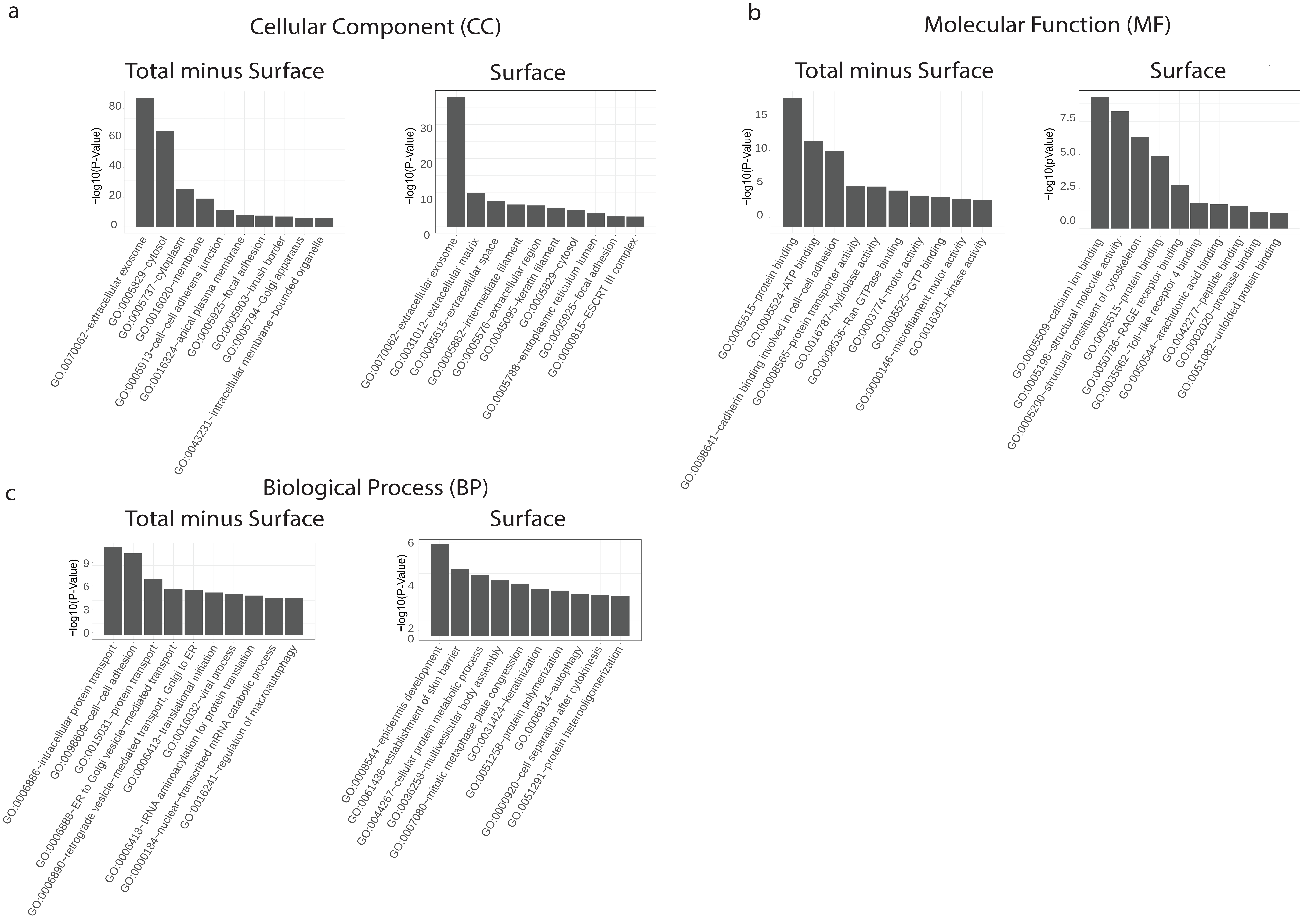
a
Cellular Component (CC)
Total minus Surface
−log10(P-Value)
0
20
40
60
80
GO:0070062~extracellular exosome
GO:0005829~cytosol
GO:0005737~cytoplasm
GO:0016020~membrane
GO:0005913~cell−cell adherens junction
GO:0016324~apical plasma membrane
GO:0005925~focal adhesion
GO:0005903~brush border
GO:0005794~Golgi apparatus
GO:0043231~intracellular membrane−bounded organelle
Surface
−log10(P-Value)
0
10
20
30
GO:0070062~extracellular exosome
GO:0031012~extracellular matrix
GO:0005615~extracellular space
GO:0005882~intermediate filament
GO:0005576~extracellular region
GO:0045095~keratin filament
GO:0005829~cytosol
GO:0005788~endoplasmic reticulum lumen
GO:0005925~focal adhesion
GO:0000815~ESCRT III complex
b
Molecular Function (MF)
Total minus Surface
−log10(P-Value)
0
5
10
15
GO:0005515~protein binding
GO:0005524~ATP binding
GO:0098641~cadherin binding involved in cell−cell adhesion
GO:0008565~protein transporter activity
GO:0016787~hydrolase activity
GO:0008536~Ran GTPase binding
GO:0003774~motor activity
GO:0005525~GTP binding
GO:0000146~microfilament motor activity
GO:0016301~kinase activity
Surface
−log10(pValue)
0.0
2.5
5.0
7.5
GO:0005509~calcium ion binding
GO:0005198~structural molecule activity
GO:0005200~structural constituent of cytoskeleton
GO:0005515~protein binding
GO:0050786~RAGE receptor binding
GO:0035662~Toll−like receptor 4 binding
GO:0050544~arachidonic acid binding
GO:0042277~peptide binding
GO:0002020~protease binding
GO:0051082~unfolded protein binding
c
Biological Process (BP)
Total minus Surface
−log10(P-Value)
0
3
6
9
GO:0006886~intracellular protein transport
GO:0098609~cell−cell adhesion
GO:0015031~protein transport
GO:0006888~ER to Golgi vesicle−mediated transport
GO:0006890~retrograde vesicle−mediated transport, Golgi to ER
GO:0006413~translational initiation
GO:0016032~viral process
GO:0006418~tRNA aminoacylation for protein translation
GO:0000184~nuclear−transcribed mRNA catabolic process
GO:0016241~regulation of macroautophagy
Surface
−log10(P-Value)
0
2
4
6
GO:0008544~epidermis development
GO:0061436~establishment of skin barrier
GO:0044267~cellular protein metabolic process
GO:0036258~multivesicular body assembly
GO:0007080~mitotic metaphase plate congression
GO:0031424~keratinization
GO:0051258~protein polymerization
GO:0006914~autophagy
GO:0000920~cell separation after cytokinesis
GO:0051291~protein heterooligomerization

Supplementary Figure 4. DAVID functional Gene Ontology analysis.

Proteins identified in the SF-sEVs fractions “Total minus Surface” and “Surface” were analyzed using DAVID Bioinformatics resources online. Enrichment analysis was performed for (a) cellular component (b) molecular function and (c) biological process. The top ten statistically significant biological terms are identified for each sample category.

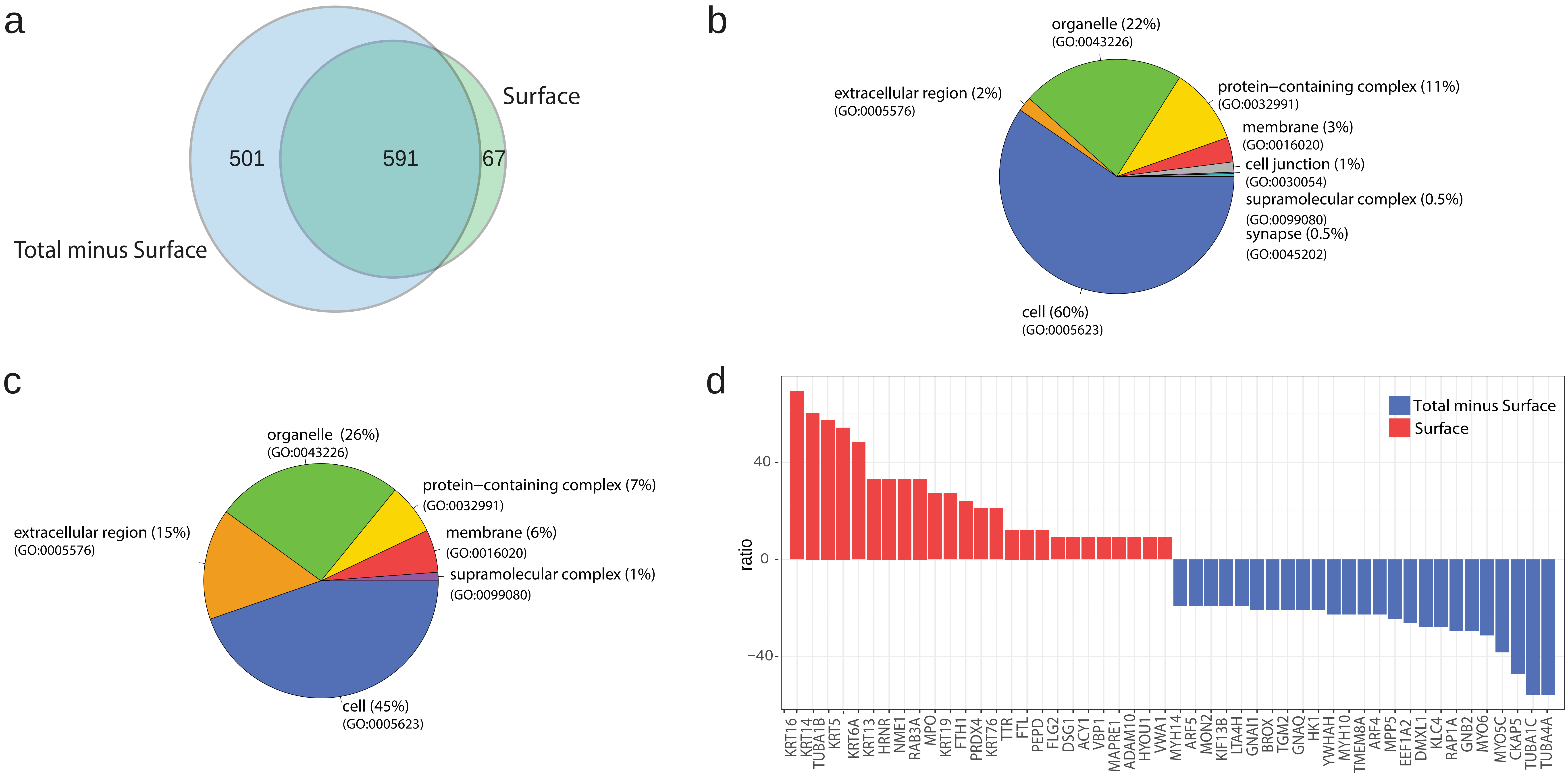
a
501
591
67
Surface
Total minus Surface
b
organelle (22%)
(GO:0043226)
extracellular region (2%)
(GO:0005576)
protein−containing complex (11%)
(GO:0032991)
membrane (3%)
(GO:0016020)
cell junction (1%)
(GO:0030054)
supramolecular complex (0.5%)
(GO:0099080)
synapse (0.5%)
(GO:0045202)
cell (60%)
(GO:0005623)
c
organelle (26%)
(GO:0043226)
protein−containing complex (7%)
(GO:0032991)
extracellular region (15%)
(GO:0005576)
membrane (6%)
(GO:0016020)
supramolecular complex (1%)
(GO:0099080)
cell (45%)
(GO:0005623)
d
Total minus Surface
Surface
ratio
40
0
−40
KRT16
KRT14
TUBA1B
KRT5
KRT6A
KRT13
HRNR
NME1
RAB3A
MPO
KRT19
FTH1
PRDX4
KRT76
TTR
FTL
PEPD
FLG2
DSG1
ACY1
VBP1
MAPRE1
ADAM10
HYOU1
VWA1
MYH14
ARF5
MON2
KIF13B
LTA4H
GNAI1
BROX
TGM2
GNAQ
HK1
YWHAH
MYH10
TMEM8A
ARF4
MPP5
EEF1A2
DMXL1
KLC4
RAP1A
GNB2
MYO6
MYO5C
CKAP5
TUBA1C
TUBA4A

Supplementary Figure 5. Protein expression in PC3 sEVs.

(a) Proportional area Venn diagram representation of proteins identified in “Surface” and “Total minus Surface” fractions. GO-CC categories of proteins enriched in (b) “Total minus Surface” and (c) “Surface” of PC3 sEVs. (d) Bar plot representing the ratio of the 25 most enriched proteins in “Surface” and in “Total minus Surface.”

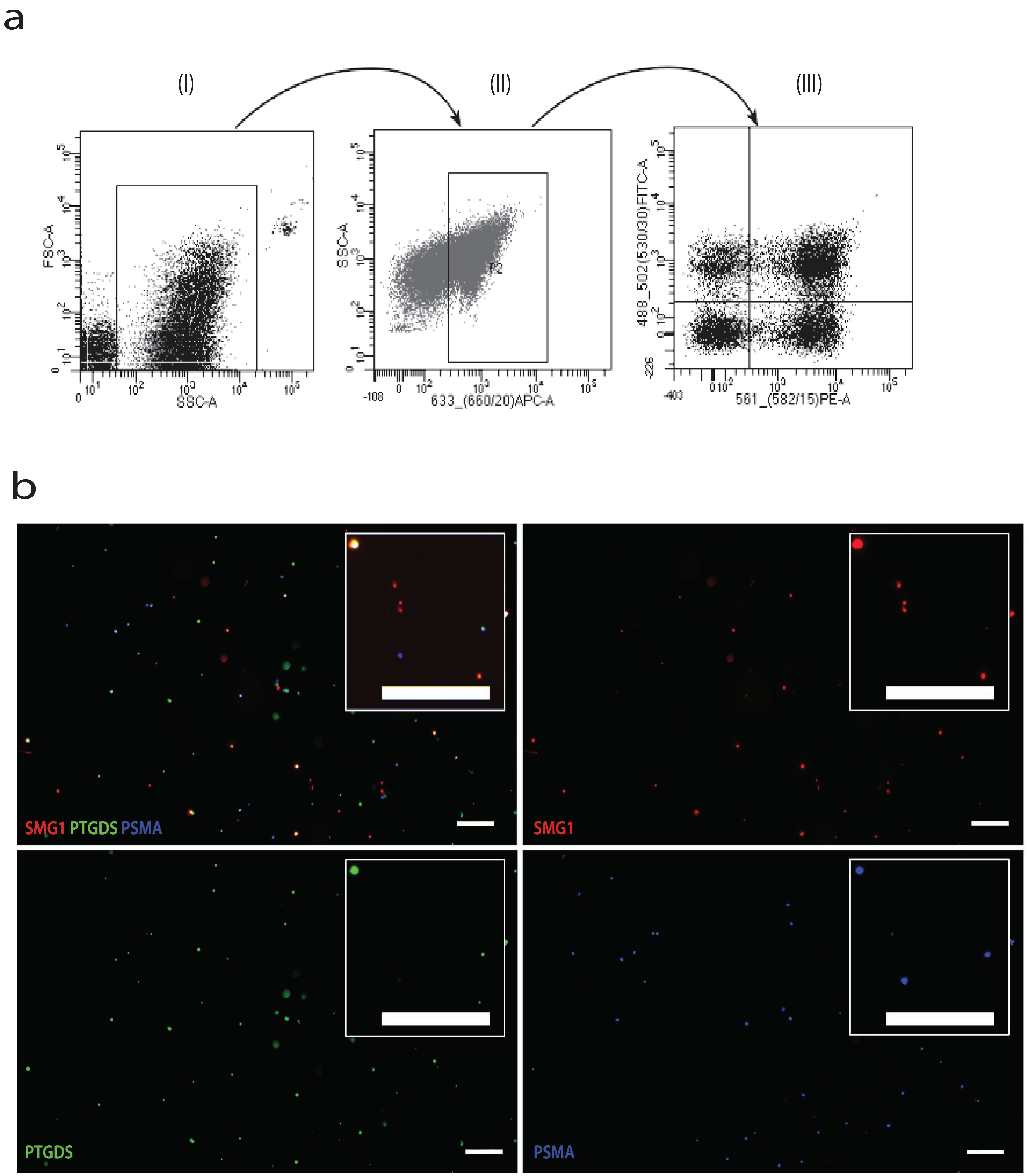
a
(I)
(II)
(III)
FSC-A
SSC-A
SSC-A
633_(660/20)APC-A
P2
488_502(530/30)FITC-A
561_(582/15)PE-A
b
SMG1 PTGDS PSMA
SMG1
PTGDS
PSMA

Supplementary Figure 6. Exo-PLA gating strategy to identify labeled sEVs.

(a) Gating of sEVs carrying RCA products; I: a gate was set around all sEVs positive for RCA products with the use of FSC/SSC and a PBS control. II: next, a gate was set around the APC positive sEVs. III: identification of the population of sEVs positive for the most abundant marker on the target sEV, followed by identifying different populations of sEVs, $FITC^{-}PE^{-}$, $FITC^{+}PE^{-}$, $FITC^{-}PE^{+}$, $FITC^{+}PE^{+}$. In this example of gating strategy APC identifies populations positive and negative for CD59, FITC and PE identify populations positive and negative for ACPP and PSMA, respectively. (b) Confirmation of positive signals by fluorescence microscopy. The images show single and triple combinations for SMG1, PTGDS and PSMA markers on single sEVs. Scale bars 20 µm

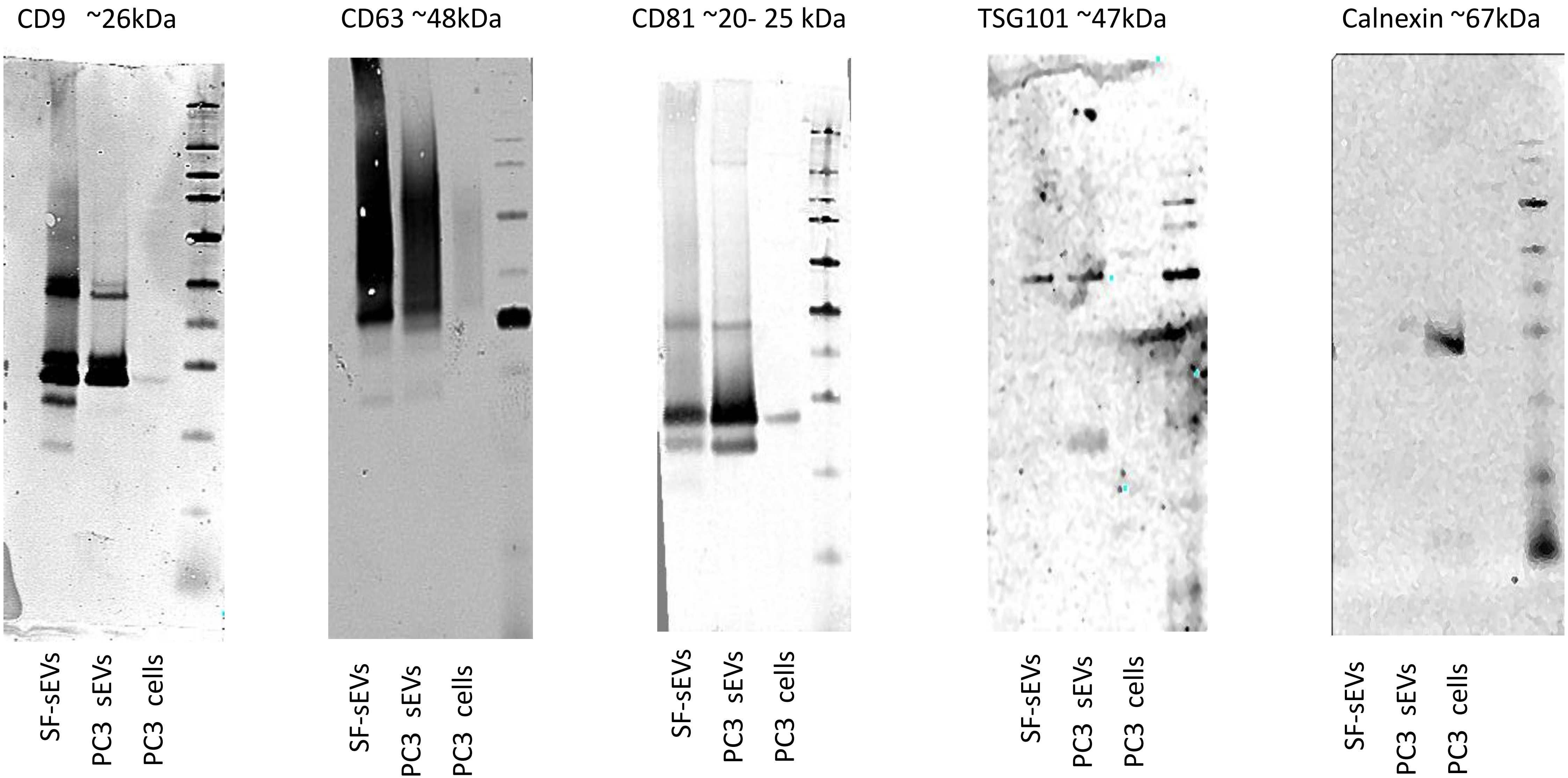
CD9 ~26kDa
CD63 ~48kDa
CD81 ~20- 25 kDa
TSG101 ~47kDa
Calnexin ~67kDa
SF-sEVs
PC3 sEVs
PC3 cells
SF-sEVs
PC3 sEVs
PC3 cells
SF-sEVs
PC3 sEVs
PC3 cells
SF-sEVs
PC3 sEVs
PC3 cells
SF-sEVs
PC3 sEVs
PC3 cells

Supplementary Figure 7. Uncropped and unedited blot western blot of seminal fluid and PC3 sEVs.

Uncropped and unedited western blot results for sEV markers CD9, CD63, CD81, TSG-101 and the ER marker calnexin was targeted as a negative control.

Supplementary Table 1. List of antibodies used in ExoPLA, SP-PLA and Western blot

Antibodies name	Target	colonality	Cat.No	Company
anti-human CD9	motility-related protein-1 (MRP-1) CD9	mAb	MAB1880-100	R&D Systems
anti-human CD63	type III lysosomal glycoprotein CD63	mAb	556019	BD Biosciences
anti-human CD81	human CD81	mAb	MAB4615	R&D Systems
anti-human CD26	Dipeptidyl peptidase-4 (DPP4) CD26	mAb	MAB1180	R&D Systems
anti-human CD13	Aminopeptidase N	mAb	MCA1270EL	AbD Serotec
anti-human ACPP	prostatic acid phosphatase	mAb	MAB6240	R&D Systems
anti-human TSG101	Tumor Susceptibility gene 101 protein	mAb	GTX70255	GeneTex
anti-human GAPDS	Glyceraldehyde-3-phosphate dehydrogenase	mAb	H00026330-M01	Abnova
anti-human PSMA	prostate-specific membrane antigen	mAb	ab19071	Abcam
anti-human Calnexin	Calnexin	mAb	ab232433	Abcam
anti-human CD9 biotinylated	motility-related protein-1 (MRP-1) CD9	mAb	13-0098-82	eBioscience
anti-human CD10	Neprilysin	pAb	AF1182	R&D Systems
anti-human PSA	Prostate-specific antigen or kallikrein-3 (KLK3)	pAb	AF1344	R&D Systems
anti-human CD59	CD59 glycoprotein	pAb	AF1987	R&D Systems
anti-human PTGDS	prostaglandin D2	pAb	orb107421	Biorbyt
anti-human AKAP 82	A-kinase anchor protein 4	pAb	GTX31595	Gene Tex
anti-human SEMG1	Semenogelin I	pAb	ABIN630160	antibodies-online.com
anti-human CRISP1	Cysteine-rich secretory protein 1	pAb	HPA028445	ATLAS ANTIBODIES
anti-human CD26 biotinylated	Dipeptidyl peptidase-4	pAb	BAF1180	R&D Systems
Secondary IRDye 680LT	donkey anti-mouse		926-68022	LI-COR
Secondary IRDye 800CW	donkey anti-rabbit		926-32213	LI-COR

Supplementary Table 2. List of oligonucleutides used in ExoPLA and SP-PLA

Name of oligos	DNA Sequence (5` to 3`)	Modification	Company
General PLA probe oligonucleotide	GACGCTAATAGTTAAGACGCTT	5’ Azide	Integrated DNA Technology
PLA probe oligonucleotide 1	AAAAAAAAAATATGACAGAACATACGGTCTCGCAGATCGCTTAGACACTCTT	5’ Azide	Integrated DNA Technology
PLA probe oligonucleotide 2	AAAAAAAAAATATGACAGAACGGACGATCATCCAGCACTAGTAGACACTCTT	5’ Azide	Integrated DNA Technology
PLA probe oligonucleotide 3	AAAAAAAAAATATGACAGAACCGGGCGACATAAGCAGATACTAGACACTCTT	5’ Azide	Integrated DNA Technology
Tag-specific 1	AGCGATCTGCGAGACCGTAT	5’phosphate	Integrated DNA Technology
Tag-specific 2	CTAGTGCTGGATGATCGTCC	5’phosphate	Integrated DNA Technology
Tag-specific 3	GTATCTGCTTATGTCGCCCG	5’phosphate	Integrated DNA Technology
Circulation oligonucleotide short	GTTCTGTCATATTTAAGCGTCTTAA	5’phosphate	Integrated DNA Technology
Circulation oligonucleotide long	CTATTAGCGTCCAGTGAATGCGAGTCCGTCTAAGAGAGTAGTACAGCAGCCGTCAAGAGTGTCTA	5’phosphate	Integrated DNA Technology
Tag-specific detection	AGCGATCTGCGAGACCGTATUUUU	5’-APC	Integrated DNA Technology
Tag-specific detection	CTAGTGCTGGATGATCGTCCUUUU	5’-FITC	Integrated DNA Technology
Tag-specific detection	GTATCTGCTTATGTCGCCCGUUUU	5’-PE	Integrated DNA Technology
Release UNG digestion oligonucleotide	AAAAACGAUUCGAGAACGUGACUGCCAUGCCAGCUCGUACUAUCGAATAATCGTACCCT	5´Azide	Integrated DNA Technology
Release UNG digestion oligonucleotide	CGAUAGUACGAGCUGGCAUGGCAGUCACGUUCUCGAAUCGUUUU	5’Biotin	Integrated DNA Technology
SLC1	CGCATCGCCCTTGGACTACGACTGACGAACCGCTTTGCCTGACTGATCGCTAAATCGTG	streptavidin-conjugated	TriLink BioTechnologies
SLC2	TCGTGTCTAAAGTCCGTTACCTTGATTCCCCTAACCCTCTTGAAAAATTCGGCATCGGTGA	streptavidin-conjugated	TriLink BioTechnologies
