# Supplementary material for: Surface protein profiling of prostate-derived extracellular vesicles by mass spectrometry and proximity assays
Source: Commun Biol. 2022 Dec 22;5:1402. doi: 10.1038/s42003-022-04349-x (PMC9780212; doi:10.1038/s42003-022-04349-x)
Supplement: Supplementary file 3 — Description of Additional Supplementary Files [file 42003_2022_4349_MOESM3_ESM.pdf]

Description of Additional Supplementary Files

File name: Supplementary Data 1
Description: A complete list of proteins identified across all the fractions for SF-sEV, PC3 sEVs and PC3 cell lysate (The source data underlying Figures 3 and 4 and Supplementary Figures 1, 2, 4 and 5)

File name: Supplementary Data 2
Description: List of proteins enriched in the surface of SF-sEVs. Only proteins identified in at least two replicates and with a ratio>2 were included. (The source data underlying Supplementary Figure 3)

File name: Supplementary Data 3
Description: Functional descriptions for The 74 surface enriched proteins
