# Supplementary material for: Surface protein profiling of prostate-derived extracellular vesicles by mass spectrometry and proximity assays
Source: Commun Biol. 2022 Dec 22;5:1402. doi: 10.1038/s42003-022-04349-x (PMC9780212; doi:10.1038/s42003-022-04349-x)
Supplement: Supplementary file 7 — Reporting Summary [file 42003_2022_4349_MOESM7_ESM.pdf]

nature portfolio

Corresponding author(s): Masood Kamali-Moghadam

Last updated by author(s): Jul 12, 2022

Reporting Summary

Nature Portfolio wishes to improve the reproducibility of the work that we publish. This form provides structure for consistency and transparency in reporting. For further information on Nature Portfolio policies, see our Editorial Policies and the Editorial Policy Checklist.

Statistics

For all statistical analyses, confirm that the following items are present in the figure legend, table legend, main text, or Methods section.

n/a	Confirmed	
☒	☐	The exact sample size (*n*) for each experimental group/condition, given as a discrete number and unit of measurement
☒	☐	A statement on whether measurements were taken from distinct samples or whether the same sample was measured repeatedly
☒	☐	The statistical test(s) used AND whether they are one- or two-sided<br>*Only common tests should be described solely by name; describe more complex techniques in the Methods section.*
☒	☐	A description of all covariates tested
☒	☐	A description of any assumptions or corrections, such as tests of normality and adjustment for multiple comparisons
☒	☐	A full description of the statistical parameters including central tendency (e.g. means) or other basic estimates (e.g. regression coefficient) AND variation (e.g. standard deviation) or associated estimates of uncertainty (e.g. confidence intervals)
☒	☐	For null hypothesis testing, the test statistic (e.g. *F*, *t*, *r*) with confidence intervals, effect sizes, degrees of freedom and *P* value noted<br>*Give P values as exact values whenever suitable.*
☒	☐	For Bayesian analysis, information on the choice of priors and Markov chain Monte Carlo settings
☒	☐	For hierarchical and complex designs, identification of the appropriate level for tests and full reporting of outcomes
☒	☐	Estimates of effect sizes (e.g. Cohen's *d*, Pearson's *r*), indicating how they were calculated

Our web collection on statistics for biologists contains articles on many of the points above.

Software and code

Policy information about availability of computer code

Data collection	*Provide a description of all commercial, open source and custom code used to collect the data in this study, specifying the version used OR state that no software was used.*
Data analysis	*Provide a description of all commercial, open source and custom code used to analyse the data in this study, specifying the version used OR state that no software was used.*

For manuscripts utilizing custom algorithms or software that are central to the research but not yet described in published literature, software must be made available to editors and reviewers. We strongly encourage code deposition in a community repository (e.g. GitHub). See the Nature Portfolio guidelines for submitting code & software for further information.

Data

Policy information about availability of data

All manuscripts must include a data availability statement. This statement should provide the following information, where applicable:

- Accession codes, unique identifiers, or web links for publicly available datasets
- A description of any restrictions on data availability
- For clinical datasets or third party data, please ensure that the statement adheres to our policy

This study includes no data deposited in external repositories.

Human research participants

Policy information about studies involving human research participants and Sex and Gender in Research.

Reporting on sex and gender	Not applicable as the study is focused on prostate.
Population characteristics	Healthy Men
Recruitment	Seminal plasma was collected at the Reproductive Centre at Uppsala University Hospital according to existing routines and under Internal Review Board authorization.
Ethics oversight	The sample collection was approved by the Ethics Committee of Uppsala University (Ups 01-367)

Note that full information on the approval of the study protocol must also be provided in the manuscript.

Field-specific reporting

Please select the one below that is the best fit for your research. If you are not sure, read the appropriate sections before making your selection.

☒ Life sciences ☐ Behavioural & social sciences ☐ Ecological, evolutionary & environmental sciences

For a reference copy of the document with all sections, see nature.com/documents/nr-reporting-summary-flat.pdf

Life sciences study design

All studies must disclose on these points even when the disclosure is negative.

Sample size	10 anonymized samples
Data exclusions	NA
Replication	samples 1 and 2 analyzed in this study were each obtained by pooling seminal plasma samples from 5 individuals.
Randomization	NA
Blinding	NA

Reporting for specific materials, systems and methods

We require information from authors about some types of materials, experimental systems and methods used in many studies. Here, indicate whether each material, system or method listed is relevant to your study. If you are not sure if a list item applies to your research, read the appropriate section before selecting a response.

Materials & experimental systems

n/a	Involved in the study	
☐	☒	Antibodies
☐	☒	Eukaryotic cell lines
☒	☐	Palaeontology and archaeology
☒	☐	Animals and other organisms
☒	☐	Clinical data
☒	☐	Dual use research of concern

Methods

n/a	Involved in the study	
☒	☐	ChIP-seq
☐	☒	Flow cytometry
☒	☐	MRI-based neuroimaging

Antibodies

Antibodies used	The antibodies, cat No, providers are listed in Supplementary Table 4
Validation	1- anti-human CD9, Cat.No MAB1880-100, https://www.rndsystems.com/products/human-cd9-antibody-209306_mab1880<br>2- anti-human CD63, Cat.No 556019, https://www.bdbiosciences.com/en-gb/products/reagents/flow-cytometry-reagents/research-reagents/single-color-antibodies-ruo/purified-mouse-anti-human-cd63.556019<br>3- anti-human CD26, Cat.No MAB1180, https://www.rndsystems.com/products/human-dppiv-cd26-antibody-222113_mab1180<br>4- anti-human CD13, Cat.No MCA1270EL, https://www.bio-rad-antibodies.com/monoclonal/human-cd13-antibody-

wm1mca1270.html?f=purified
5- anti-human ACPP, Cat.No MAB6240, https://www.rndsystems.com/products/human-prostatic-acid-phosphatase-acpp-antibody-690017_mab6240
6- anti-human TSG101 , Cat.No GTX70255, https://www.genetex.com/Product/Detail/TSG101-antibody-4A10/GTX70255
7- anti-human GAPDS, Cat.No H00026330-M01, http://www.abnova.com/products/products_detail.asp?catalog_id=H00026330-M01
8- anti-human PSMA, Cat.No ab19071, Anti-PSMA antibody [YPSMA-1] (ab19071) is not available any more
9- anti-human Calnexin, Cat.No ab232433, https://www.abcam.com/calnexin-antibody-epr3632-bsa-and-azide-free-ab232433.html
10- anti-human CD9 biotinylated, Cat.No 13-0098-82, https://www.thermofisher.com/antibody/product/CD9-Antibody-clone-eBioSN4-SN4-C3-3A2-Monoclonal/13-0098-82
11- anti-human CD10, Cat.No AF1182, https://www.rndsystems.com/products/human-neprilysin-cd10-antibody_af1182
12- anti-human PSA, Cat.No AF1344, https://www.rndsystems.com/products/human-kallikrein-3-psa-antibody_af1344
13- anti-human CD59, Cat.No AF1987, https://www.rndsystems.com/products/human-cd59-antibody_af1987
14- anti-human PTGDS, Cat No orb107421, https://www.biorbyt.com/ptgds-antibody-orb107421.html
15- anti-human AKAP 82, Cat No GTX31595, https://www.genetex.com/Product/Detail/AKAP-82-antibody/GTX31595
16- anti-human SEMG1, Cat No ABIN630160, https://www.antibodies-online.com/antibody/630160/anti-Semenogelin+I+SEMG1+N-Term+antibody/
17- anti-human CRISP1, Cat No HPA028445, https://nordicbiosite.com/product/HPA028445-100/CRISP1
18- anti-human CD26 biotinylated, Cat No BAF1180, https://www.rndsystems.com/products/human-dppiv-cd26-biotinylated-antibody_baf1180
19- Secondary IRDye 680LT, Cat No 926-68022, https://www.licor.com/bio/reagents/irdye-680lt-donkey-anti-mouse-igg-secondary-antibody
20- Secondary IRDye 800CW, Cat.No 926-32213, https://www.licor.com/bio/reagents/irdye-800cw-donkey-anti-rabbit-igg-secondary-antibody

Eukaryotic cell lines

Policy information about cell lines and Sex and Gender in Research

Cell line source(s)	PC3 cell line, Human cells, epithelial , Prostate, Adenocarcinoma; Grade IV, provider :ATCC website
Authentication	Cell line did not authenticated by researcher but informations provide by ATCC
Mycoplasma contamination	We confirmed negative Mycoplasma contamination.
Commonly misidentified lines (See ICLAC register)	*Name any commonly misidentified cell lines used in the study and provide a rationale for their use.*

Flow Cytometry

Plots

Confirm that:

☒ The axis labels state the marker and fluorochrome used (e.g. CD4-FITC).

☒ The axis scales are clearly visible. Include numbers along axes only for bottom left plot of group (a 'group' is an analysis of identical markers).

☒ All plots are contour plots with outliers or pseudocolor plots.

☐ A numerical value for number of cells or percentage (with statistics) is provided.

Methodology

Sample preparation	It has been describe in details in Material and Methods section.
Instrument	BD FACS Aria III or BD LSR Fortessa instruments (BD biosciences)
Software	Data analysis was performed using BD FACS Diva software 8.0 (BD biosciences)
Cell population abundance	NA
Gating strategy	Gating of sEVs carrying RCA products; I: a gate was set around all sEVs positive for RCA products with the use of FSC/SSC and a PBS control. II: next, a gate was set around the APC positive sEVs. III: identification of the population of sEVs positive for the most abundant marker on the target sEV, followed by identifying different populations of sEVs, FITC-PE-, FITC+PE-, FITC-PE+, FITC+PE+. In this example of gating strategy APC identifies populations positive and negative for CD59, FITC and PE identify populations positive and negative for ACPP and PSMA, respectively. (b) Confirmation of positive signals by fluorescence microscopy. The images show single and triple combinations for SMG1, PTGDS and PSMA markers on single sEVs. Scale bars 20 µm.

☒ Tick this box to confirm that a figure exemplifying the gating strategy is provided in the Supplementary Information.
